# Estrogen Influences Human Microvascular Endothelial Function Via Sex-Specific Regulation of Sphingolipids

**DOI:** 10.1016/j.jacbts.2025.101389

**Published:** 2025-10-25

**Authors:** Gopika SenthilKumar, Zachary Zirgibel, Maria J. Jaramillo-Torres, Rachel H. Limpert, Katie E. Cohen, Carolyn Shult, Brian Lindemer, Henry Bordas-Murphy, Callisia N. Clarke, Julie K. Freed

**Affiliations:** a Department of Anesthesiology, Medical College of Wisconsin, Milwaukee, Wisconsin, USA; b Department of Physiology, Medical College of Wisconsin, Milwaukee, Wisconsin, USA; c Cardiovascular Center, Medical College of Wisconsin, Milwaukee, Wisconsin, USA; d Division of Cardiovascular Medicine, Department of Medicine, Medical College of Wisconsin, Milwaukee, Wisconsin, USA; e Division of Surgical Oncology, Department of Surgery, Medical College of Wisconsin, Milwaukee, Wisconsin, USA

**Keywords:** ceramide, endothelial dysfunction, estrogen, microvasculature

## Abstract

Microvascular endothelial dysfunction strongly predicts poor cardiovascular outcomes, and recent studies have indicated that chronic estrogen treatment promotes endothelial dysfunction in isolated human microvessels. Here, using a translational approach, we demonstrate that sphingolipids are a critical component of estrogen signaling within the human microvascular endothelium. We provide evidence that the stark differences in how estrogen influences the microvascular endothelium are both time- and sex-dependent. This work adds mechanistic insight regarding the complexity and often contradictory relationship between estrogen and cardiovascular risk in humans.

## Highlights

• Chronic estrogen exposure promotes sex-specific human microvascular endothelial dysfunction in isolated arterioles ex vivo; however, mechanisms remain unknown.
• Estrogen can stimulate the NSmase pathway to generate endothelial ceramide in a sex-dependent manner.
• A thorough understanding of how estrogen regulates the human microvascular endothelium will provide needed insight for combatting coronary microvascular dysfunction, a disease more prevalent in women.

## Visual Abstract

**Figure:**
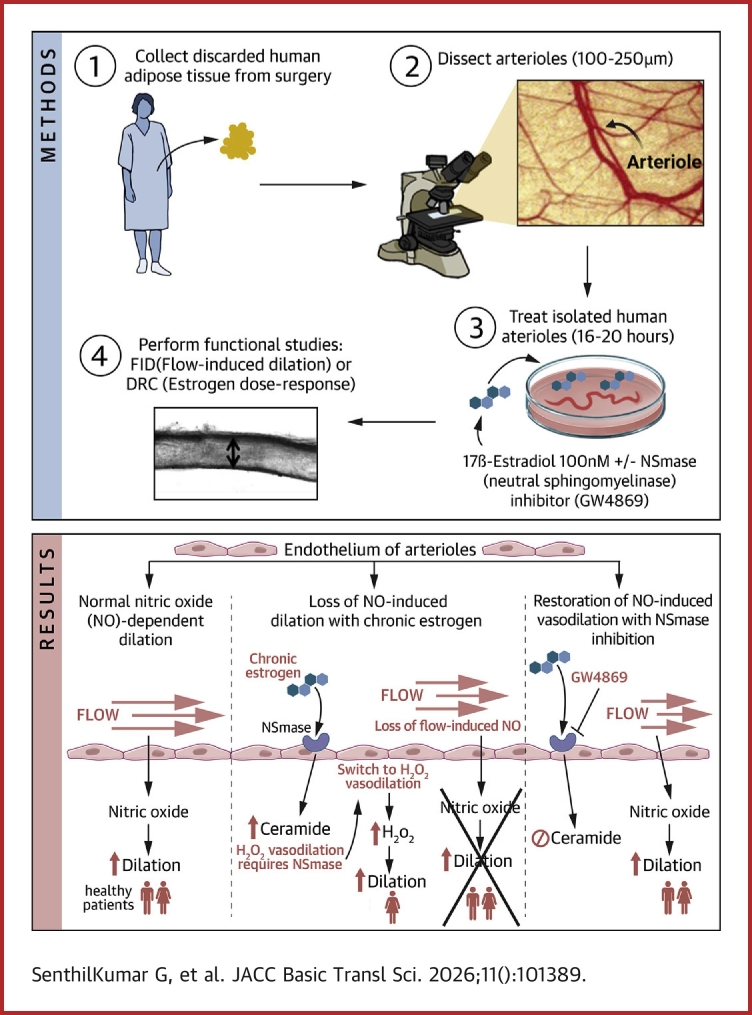

Although estrogen has historically been regarded as a cardioprotective hormone, recent studies challenge this paradigm by linking estrogen with detrimental cardiovascular effects. Chronic estrogen use for contraception,^1^^,^^2^ management of menopausal symptoms,^3^^,^^4^ and gender affirmation^5^, ^6^, ^7^, ^8^ is associated with increased risk for cardiovascular disease (CVD). Our recent work has shown that chronic exposure of human arterioles ex vivo to 100 nmol/L 17β-estradiol (E2), a dose known to elicit vasculoprotection in preclinical studies, can trigger microvascular endothelial dysfunction in microvessels from otherwise healthy adults.^9^ This presented as a switch in the mediator of flow-induced dilation (FID) from vasoprotective nitric oxide (NO) to proinflammatory hydrogen peroxide (H_2_O_2_) in vessels from healthy women regardless of age. Interestingly, arterioles from healthy men and a subset of women exhibited severe endothelial dysfunction following estrogen treatment as evidenced by a stark reduction in vasodilatory capacity to flow.^9^ Contrary to these findings, ample preclinical evidence exists indicating that estrogen can promote endothelial NO production and suppress oxidative stress.^10^, ^11^, ^12^, ^13^ The mechanism that may be driving these paradoxical effects of estrogen remain unknown. It is theorized that estrogen may acutely promote beneficial NO signaling because cardioprotection is linked to women with naturally cycling estrogen levels as well as in preclinical studies that demonstrate increases in NO following a brief exposure. On the contrary, these acute benefits may not be sustained with chronic administration of estrogen, resulting in poor cardiovascular outcomes among women on long-term oral contraceptives/menopausal hormone therapy or women of trans experience undergoing gender-affirming therapy.^14^

Estrogen is a well-established modulator of sphingolipid signaling and can increase endothelial expression of the flow-sensitive, ceramide-forming enzyme neutral sphingomyelinase (NSmase).^15^^,^^16^ Further, acute estrogen signaling (nongenomic) is known to activate the PI3K/MAPK pathway, which can subsequently stimulate NSmase.^17^, ^18^, ^19^ Although acute NSmase activation and ceramide production is necessary for maintaining NO-mediated dilation to flow as well acetylcholine of arterioles from otherwise healthy adults,^19^^,^^20^ chronic NSmase activation and ceramide accumulation promotes microvascular endothelial dysfunction.^20^, ^21^, ^22^ Mechanistically, acute ceramide exposure stimulates the generation of NO via its conversion to sphingosine-1-phosphate (S1P) within seconds to minutes, while chronic accumulation of ceramide is associated with impaired S1P signaling and increased mitochondrial H_2_O_2_ production.^19^^,^^20^^,^^23^^,^^24^ Furthermore, acute estrogen administration can activate sphingosine kinase, the rate-limiting step to convert ceramide to S1P.^25^ Here, we hypothesized that the immediate NO-generating effect of estrogen is caused by stimulation of the NSmase/ceramide/S1P pathway, while longer exposure results in ceramide accumulation and mitochondrial dysfunction. We evaluated this hypothesis using peripheral human arterioles as well as sex-specific isolated human endothelial cells. To our knowledge, this is the first translational study to identify a unifying mechanism that explains how estrogen is capable of exerting both beneficial (acute NO signaling) and detrimental (endothelial dysfunction) effects in the human microvasculature, a vascular bed that when dysfunctional, highly predicts poor cardiovascular outcomes.^26^

## Methods

### Human tissue collection

All protocols utilized were approved by the Institutional Review Board at the Medical College of Wisconsin. Fresh adipose tissue otherwise discarded from surgery was acquired and placed in cold 4-(2-hydroxyethyl)-1-piperazineethanesulfonic acid (HEPES) buffer (4 °C). Patient data (age, biological sex, known risk factors for coronary artery disease, prescription of medications for cardiovascular diseases) devoid of personal identifiers were retrieved from the REDCap database housed at the Medical College of Wisconsin. When possible, informed consent was obtained from patients scheduled for surgeries expected to yield discardable tissue to allow access to more extensive past medical history information. Patients were considered “healthy” if they had 0 to 1 risk factor(s) for coronary artery disease, including hypertension, active smoking, hyperlipidemia, congestive heart failure, and diabetes mellitus (type 1 or 2). Across both methods of tissue collection, we received information on patient age, body mass index (BMI), biological sex, race, and the risk factors listed in the previous text.

### Human microvascular function studies

Human arterioles were dissected from the discarded surgical adipose tissue and washed overnight in HEPES buffer (4 °C) before cannulation for pressure myography. Vessels were then cannulated onto glass micropipettes of matched impedance within an organ chamber containing KREBs buffer with a controlled pH of 7.4 and temperature of 37 °C. Fluid reservoirs with KREBs were connected to the pipettes to establish arteriolar pressures and microvessels were equilibrated at 30 mm Hg followed by 60 mm Hg for 30 minutes each. During pressurization at 60 mm Hg, treatments (Table 1) were added. Endothelin-1 (Sigma, E7764-50UG, 0.2-2 nmol/L) was used to preconstrict arterioles to 30% to 75% of their passive diameters. Microvessels that did not maintain constriction (<5% change in constricted diameter for 10 minutes) were deemed nonviable. To assess the response to acute exogenous estrogen, increasing doses (10^−11^ to 10^−5^ mol/L) of E2 were administered in 2-minute intervals followed by measurement of internal arteriolar diameter using video microscopy. To assess dilation to flow, a pressure gradient was induced by moving the fluid reservoirs in equal and opposite directions and changes in the internal diameters were measured after 5-minute exposure to each pressure gradient (5 to 100 cmH_2_O). This protocol was originally described by Kuo et al,^27^ and has been extensively used in our laboratory.^9^^,^^20^, ^21^, ^22^ Two flow response experiments were conducted; the first to assess overall capacity to dilate to flow, and the second to determine the effect of added inhibitors. In the event a vessel exhibited reduced vasodilatory capacity during the first experiment, a second flow curve was not generated. Following each flow experiment, arterioles were exposed to 100 μmol/L papaverine (endothelial-independent dilator; Sigma P3510) to assess smooth muscle integrity. Percent change in arteriolar diameter was calculated with 100% representing the vessel’s maximal diameter and 0% representing the endothelin-1 preconstricted diameter. A detailed description of this methodology as well as rationale for the methodology utilized for calculating maximal dilation to flow can be found here.^20^

**Table 1:** Treatment Details

Treatment	Description	Concentration
17β-estradiol	Estrogen	10^−11^ to 10^−5^ mol/L
GW4869	Neutral sphingomyelinase inhibitor	4 μmol/L
2-(4-carboxyphenyl)-4,4,5,5-tetramethylimidazoline-1-oxyl-3-oxide (cPTIO)	Nitric oxide scavenger	100 μmol/L
Nω-nitro-L-arginine methyl ester (L-NAME)	Nitric oxide synthase inhibitor	100 μmol/L
Polyethylene glycol-catalase (catalase)	Enzyme that catabolizes H_2_O_2_	500 U
SpK-I	Sphingosine-kinase inhibitor	1 μmol/L
W146	S1P-receptor-1 inhibitor	10 μmol/L
MitoTempol	Mitochondria-targeted antioxidant	100 μmol/L
GSK2795039	NADPH-oxidase 2 inhibitor	1 μmol/L

### Cell culture

Human umbilical vein endothelial cells (ATCC Manassas) from 3 women and 3 men were pooled by biological sex and cultured using 5% fetal bovine serum containing endothelial cell growth medium (EBM-2 Basal Medium; Lonza CC-3156). Only cell passage numbers 2 to 5 were utilized. Cells were plated at 50% to 60% confluency onto 8-well chamber slides (Ibidi) for H_2_O_2_ measurement or onto 10-cm dishes for cell collection.

### Measurement of H_2_O_2_ in cultured endothelial cells

Endothelial cells seeded in 8-well chamber slides were treated for 48 hours with estrogen +/− treatments or ceramide as described in results. Peroxy yellow 1 probe (Sigma SML0676; diluted in phosphate-buffered saline [PBS]; Invitrogen A14291DJ) was added to each well for 1 hour (25 μmol/L) and images were acquired using a Keyence fluorescence microscope (488 per 510 nm excitation/emission) at 4 different imaging fields per well. The same exposure settings were used across all wells. The mean fluorescence intensity per image was calculated using ImageJ and the percent change in intensity from the average control (untreated cells) intensity for a given exposure was calculated for all images. This value was averaged per well. Pegylated-catalase (catalase, 500 U) was added to confirm signal specificity for H_2_O_2_.

### High-performance liquid chromatography and tandem mass spectrometry

Sex-specific endothelial cells were seeded in 10-cm dishes and treated for 48 hours with 1 nmol/L E2, 100 nmol/L E2, or vehicle control (EtOH %vol/vol). Cells were then trypsinized, added into 5-mL tubes which contained 4 mL of media to deactivate the trypsin, and centrifuged at 1,500 RPM for 5 minutes to form a cell pellet. The media/trypsin mixture was removed, and 2 mL PBS was added for washing. The tube was centrifuged again with the same settings, PBS was removed, and the cell pellets were frozen at −80 °C.

Sphingolipid levels were measured by high-performance liquid chromatography and tandem mass spectrometry as previously described at the Medical University of South Carolina Lipidomics Shared Resource Core.^28^, ^29^, ^30^ A Thermo Scientific Vanquish uHPLC system coupled to a Thermo Scientific Quantum Access Max triple quadruple mass spectrometer equipped with an ESI probe operating in the multiple reaction monitoring positive ion mode was used to analyze the samples. Chromatographic separations were obtained under a gradient elution on a C8 column using a mobile phase with ammonium formate, formic acid in water, and methanol. Prior to analysis samples underwent an ethyl acetate/isopropanol liquid-liquid extraction.^31^ Quantitative analyses of sphingolipids were based on 8-point calibration curves generated for each target analyte. The synthetic standards along with a set of internal standards were spiked into an artificial matrix and were then subject to an identical extraction procedure as the biological samples. These extracted standards were then analyzed with the samples by the high-performance liquid chromatography and tandem mass spectrometry. Peaks for the target analytes and internal standards were recorded and processed using the instrument’s software. Analyte/internal standard peak area ratios were plotted against analyte concentrations to generate the sphingolipid specific calibration curves. Any sphingolipids for which no standards were available were quantitated using the calibration curve of its closest counterpart. Sphingolipid levels were normalized to total inorganic phosphate and expressed as picomoles per nanomole of inorganic phosphate.

### Statistical analysis

Vascular data are presented using the mean ± SEM. Patient demographics of biological men and women were compared using a 2-tailed Student’s *t*-test for continuous variables or chi-square analysis for categorical variables. Normalized endothelial sphingolipid levels as well as endothelial H_2_O_2_ fluorescence were compared using 2-tailed Student’s *t*-test (2 groups) or 1-way analysis of variance (2+ groups). Dilation to increasing pressure gradients or E2 doses was compared between groups using a 2-way repeated-measures analysis of variance with pressure gradients/estrogen doses and treatments as parameters. When multiple comparisons were performed, Holm-Sidak post hoc test for multiple pairwise comparisons was applied. Normality of the data distributions was evaluated using the Shapiro-Wilk test. Statistical significance was set at *P <* 0.05, and all analyses were all performed using GraphPad Prism version 9.5 (GraphPad Software).

### Data availability

The authors declare that all data supporting the findings of this study are available within the article and its supplementary information.

## Results

A total of 43 patients were included: 9 men and 34 women. Average age (mean ± SEM) of men was 48.0 ± 4.5 and women was 39.6 ± 2.2 years *(P =* 0.093), and BMI of men was 28.6 ± 1.9 and women was 29.1 ± 1.1 kg/m^2^ *(P =* 0.828). Among the men, 7 identified as White and 2 identified as Black, while among the women, 23 identified as White, 7 identified as Black, 1 as Asian, and 3 as Hispanic. Risk factors among the patients included hypertension (n = 3), diabetes (n = 2), and smoking (n = 3). The remaining patients had no known risk factors for coronary artery disease.

### Estrogen acutely promotes NO-mediated dilation of human arterioles via NSmase/ceramide/S1P signaling

**Figure 1:**
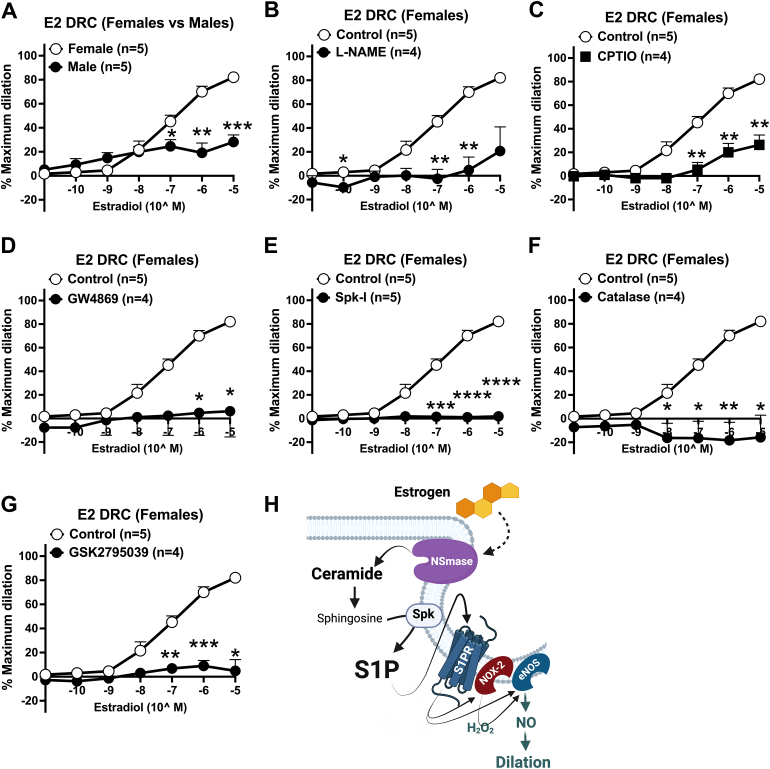
Estrogen Elicits Acute NO-Mediated Dilation of Human Arterioles Via Ceramide/S1P Signaling (A) Dilation to 17β-estradiol (E2) (10^−11^ to 10^−5^, 2 minutes) in control vessels from healthy women (n = 5) vs men (n = 5). Dilation to increasing doses of E2 in vessels from healthy women treated with 30 minutes of (B) nitric oxide synthase inhibitor L-NAME (100 μmol/L; n = 4), (C) NO scavenger CPTIO (100 μmol/L; n = 4), (D) NSmase inhibitor GW4869 (4 μmol/L; n = 4), (E) sphingosine-kinase inhibitor Spk-I (1 μmol/L; n = 5), (F) catalase (500 U/ml; n = 4), (G) NADPH-oxidase-2-inhibitor GSK2795039 (1 μmol/L; n = 4). (H) Schematic representation of results created with Biorender.com. Data are presented as mean ± SEM. Two-way repeated measures analysis of variance with Holm-Sidak multiple comparisons test. ∗*P <* 0.05, ∗∗*P <* 0.01, ∗∗∗*P <* 0.001.

We first assessed whether increasing doses of E2 (10^−11^ to 10^−5^ mol/L, 2 minutes) induces vasodilation of resistance arterioles from otherwise healthy women and men. Interestingly, only arterioles from women exhibited significant dilation to increasing doses of estrogen (Figure 1A) compared with men. Vasodilation of arterioles from healthy women was diminished in the presence of the NO synthase inhibitor L-NAME (100 μmol/L, 30 minutes) (Figure 1B) as well as the NO scavenger CPTIO (100 μmol/L, 30 minutes) (Figure 1C). To evaluate whether estrogen-induced NO production in female arterioles requires NSmase, estrogen was administered in the presence of the NSmase inhibitor GW4869 (4 μmol/L, 30 minutes) which led to diminished dilation to E2 (Figure 1D). Dilation to E2 was also significantly reduced in the presence of a sphingosine-kinase inhibitor (SpK-I, 1 μmol/L, 30 minutes) (Figure 1E). Prior studies have indicated a necessary role of H_2_O_2_ production during activation of the ceramide/S1P/NO pathway, primarily from NADPH-oxidase-2.^20^^,^^32^ As shown in Figures 1F and 1G, treatment with catalase (500 U, 30 minutes) as well as an inhibitor of NADPH-oxidase-2 GSK2795039 (1 μmol/L, 30 minutes) impaired dilation to E2. The role of ceramide/S1P signaling in promoting estrogen-induced NO production is schematized in Figure 1H.

### Chronic estrogen promotes microvascular dysfunction in human arterioles via NSmase/ceramide/H_2_O_2_ signaling

**Figure 2:**
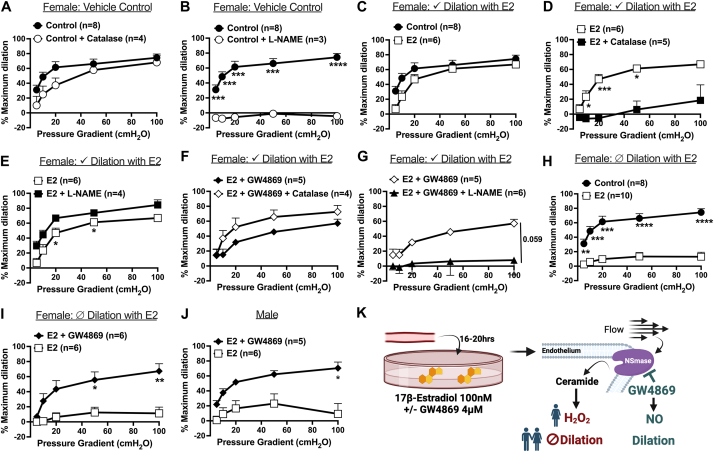
NSmase Inhibition Prevents Chronic Estrogen-Induced Human Microvascular Dysfunction in Women and Men Flow-induced dilation in vessels from women treated with vehicle (EtOH, %vol/vol; 16-20hrs; n = 8) in the presence of (A) catalase (500U/ml; n = 4) and (B) L-NAME (100 μmol/L; n = 3). (C) flow-induced dilation in vessels from women treated with vehicle control (n = 8) versus 100 nmol/L 17β-estradiol (E2;16-20hrs) and able to maintain dilation to flow >50% of max dilation (n = 6). flow-induced dilation in E2 treated vessels (n = 6) in the presence of (D) catalase (n = 5) and (E) L-NAME (n = 4). flow-induced dilation in vessels from women treated with E2 + GW4869 (4 μmol/L; n = 4) in the presence of (F) catalase (n = 4) and (G) L-NAME (n = 6). (H) flow-induced dilation in vessels from women treated with vehicle control (n = 8) versus those treated with E2 and had impaired flow-induced dilation (<50% of max dilation; n = 10). (I) flow-induced dilation in E2 treated vessels (n = 6) with GW4869 (n = 6). (J) flow-induced dilation in vessels from men treated with E2 (n = 6) versus E2 +GW4869 (n = 5). (K) Schematic of results. Data are presented as mean ± SEM. Two-way repeated measures analysis of variance with Holm-Sidak multiple comparisons test ∗*P <* 0.05, ∗∗*P <* 0.01, ∗∗∗*P <* 0.001.

We next evaluated whether estrogen-induced microvascular dysfunction from chronic exposure (100 nmol/L, 16-20 hours) was dependent on activation of NSmase. Arterioles from otherwise healthy women exhibited NO-mediated dilation following vehicle control treatment, as catalase did not impair dilation to flow (Figure 2A) as opposed to treatment with L-NAME (Figure 2B). Dilation to flow was maintained following E2 treatment in arterioles from a cohort of healthy women (Figure 2C); however, a switch in FID mediator to H_2_O_2_ was observed as dilation to flow was impaired during exposure to catalase (Figure 2D), whereas L-NAME had no effect (Figure 2E). Exposure to the NSmase-inhibitor GW4869 (16-20 hours) during E2 treatment prevented dependency on H_2_O_2_ for dilation and may promote NO-mediated dilation (Figures 2F and 2G). Arterioles from a cohort of otherwise healthy women portrayed diminished dilation to flow following E2 exposure (Figure 2H), which was restored with simultaneous treatment with GW4869 (Figure 2I). No significant differences in age or BMI were observed among those that maintained vs did not maintain dilation to flow following chronic E2 treatment (no dilation vs dilation mean ± SE; age: 44.9 ± 4.1 years vs 44.7 ± 3.8 years; *P =* 0.97; BMI: 29.0 ± 2.4 kg/m^2^ vs 28.1 ± 2.1 kg/m^2^; *P =* 0.79). A decreased response to flow was noted in arterioles from men following chronic exposure to E2, an effect that was prevented by NSmase inhibition (Figure 2J). These results, showing that inhibition of NSmase-mediated ceramide formation can prevent chronic estrogen-mediated vascular damage regardless of whether vessels maintained dilation to flow after estrogen exposure, are summarized by the schematic in Figure 2K.

### Chronic estrogen administration impairs acute estrogen signaling via NSmase and promotes H_2_O_2_-mediated dilation in response to acute ceramide

**Figure 3:**
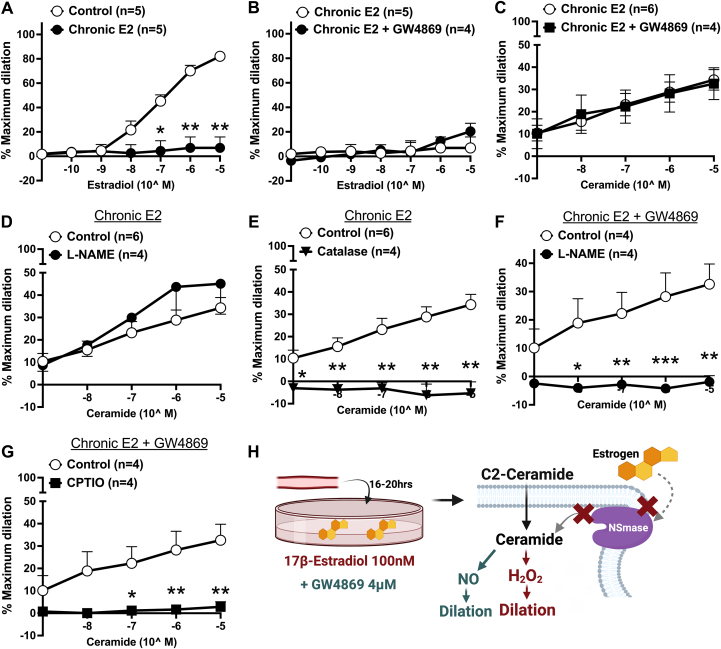
Chronic Estrogen Administration Impairs Acute Estrogen-Signaling iIn Arterioles From Women and Alters Ceramide Signaling to Promote H_2_O_2_ as Opposed to NO Production Dilation to 17β-estradiol (E2; 10^−11^ to 10^−5^, 2 minutes) in vessels from women treated with 100 nmol/L E2 for 16 to 20 hours (n = 5) compared with (A) untreated vessels (n = 5) and (B) vessels treated with 100 nmol/L E2 + NSmase-inhibitor GW4869 (n = 4; 4 μmol/L; 16-20 hours). (C) Dilation to C2-ceramide (10^−9^ to 10^−5^, 1 minute) in vessels from women treated with 16-20 hours E2 (n = 6) or E2+GW4869 (n = 4). Within vessels treated with 16 to 20 hours of E2 (n = 6), dilation to ceramide in the presence of (D) L-NAME (100 μmol/L; n = 4) or (E) catalase (500 U/ml, n = 4). Within vessels treated with 16 to 20 hours of E2+GW4869 (n = 4), dilation to ceramide in the presence of (F) L-NAME (n = 4) or (G) CPTIO (n = 4). (H) Schematic representation of results created with Biorender.com. Data are presented as mean ± SEM. Two-way repeated measures analysis of variance with Holm-Sidak multiple comparisons test. ∗*P <* 0.05, ∗∗*P <* 0.01, ∗∗∗*P <* 0.001.

We next evaluated whether chronic E2 exposure affects the integrity of the acute E2/NSmase/NO signaling pathway. As shown in Figure 3A, following chronic E2 treatment, acute estrogen-induced vasodilation was reduced. This response was maintained despite treatment with GW4869 (Figure 3B), suggesting that the loss of E2/NSmase/NO signaling is independent of activation of NSmase. To ensure the change in estrogen signaling was specific to the NSmase pathway, dilation to another endothelial-dependent mediator (acetylcholine) was tested following chronic E2 which was preserved (Supplemental Figure 1).

Prior work indicates that acute exposure to ceramide results in a modest dilation in arterioles from healthy adults caused by formation of NO^20^; therefore, we explored whether downstream ceramide signaling remained intact following chronic E2 exposure. Although microvessels maintained the ability to dilate (Figure 3C), they transitioned to H_2_O_2_-dependent FID (Figures 3D and 3E), an effect that was preventable by inhibiting NSmase with GW4869 (Figures 3F and 3G). Figure 3H schematizes these results.

### Chronic estrogen-induced endothelial dysfunction is associated with accumulation of intracellular ceramides

**Figure 4:**
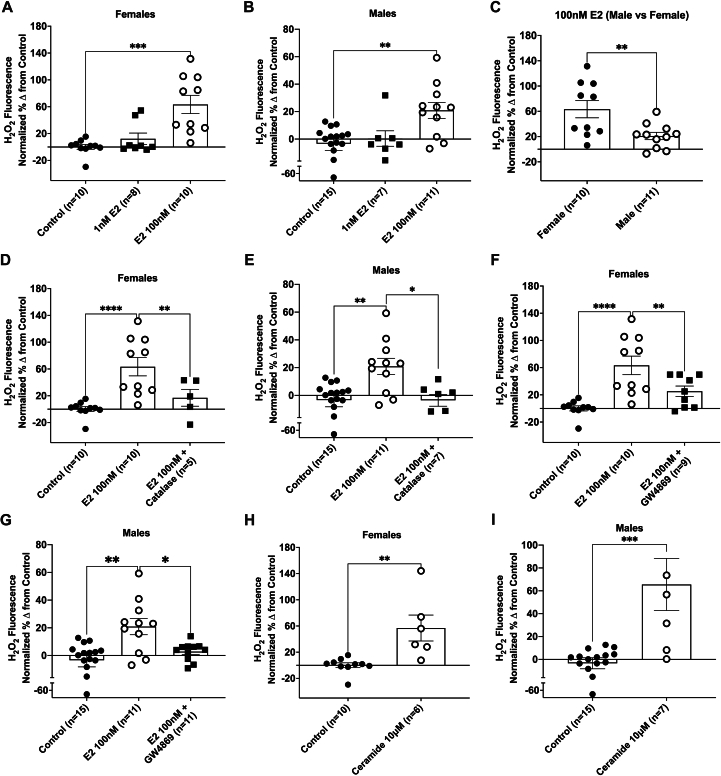
Chronic Estrogen Exposure Results in Ceramide Accumulation and Promotes the Formation of Endothelial H_2_O_2_ Formation H_2_O_2_ fluorescence, as measured using peroxy-yellow-1 (PY1) in human umbilical vein endothelial cells treated with 1 and 100 nmol/L 17β-estradiol (E2) (48 hours) in (A) women (control n = 10, 1 nmol/L E2 n = 8, 100 nmol/L E2 n = 10) and (B) men (control n = 15, 1 nmol/L E2 n = 7, 100 nmol/L E2 n = 11); (C) Sex difference. H_2_O_2_ in endothelial cells treated with 100 nmol/L E2 +/− catalase (500 U/ml) in (D) women (control n = 10, E2 n = 10, E2+catalase n = 7) and (E) men (control n = 15, E2 n = 11, E2+catalase n = 5). H_2_O_2_ production in the presence of E2 vs E2+ GW4869 (4 μmol/L; 48 hours) in endothelial cells from (F) women (control n = 10, E2 n = 10, E2+GW4869 n = 9) and (G) men (control n = 15, E2 n = 11, E2+GW4869 n = 11). Endothelial H_2_O_2_ following exposure to C2-ceramide (10 μmol/L, 48 hours) in (H) women (control n = 10, ceramide n = 6) and (I) men (control n = 15, ceramide n = 7). Data are presented as mean ± SEM. (A, B, D-G) 1-way analysis of variance with Holm-Sidak multiple comparisons test and (C, H-I) 2-tailed *t*-test. ∗*P <* 0.05, ∗∗*P <* 0.01, ∗∗∗*P <* 0.001.

We utilized sex-specific human umbilical vein endothelial cells (HUVECs) to confirm that chronic E2 exposure promotes *endothelial-specific* dysfunction via NSmase-dependent ceramide formation. Treatment of endothelial cells with 48 hours of 100 nmol/L E2 resulted in a significant increase in H_2_O_2_ production in cells from women (Figure 4A) and men (Figure 4B), an effect more robust in women compared with men (Figure 4C). Treatment with catalase prevented this increase, confirming signaling specificity for H_2_O_2_ (Figures 4D and 4E). Inhibition of NSmase with GW4869 prevented estrogen-induced H_2_O_2_ production in endothelial cells from both women (Figure 4F) and men (Figure 4G). Exposure to exogenous C2-ceramide (10 μmol/L, 48 hours) increased endothelial H_2_O_2_ production in endothelial cells from both sexes (Figures 4H and 4I).

**Figure 5:**
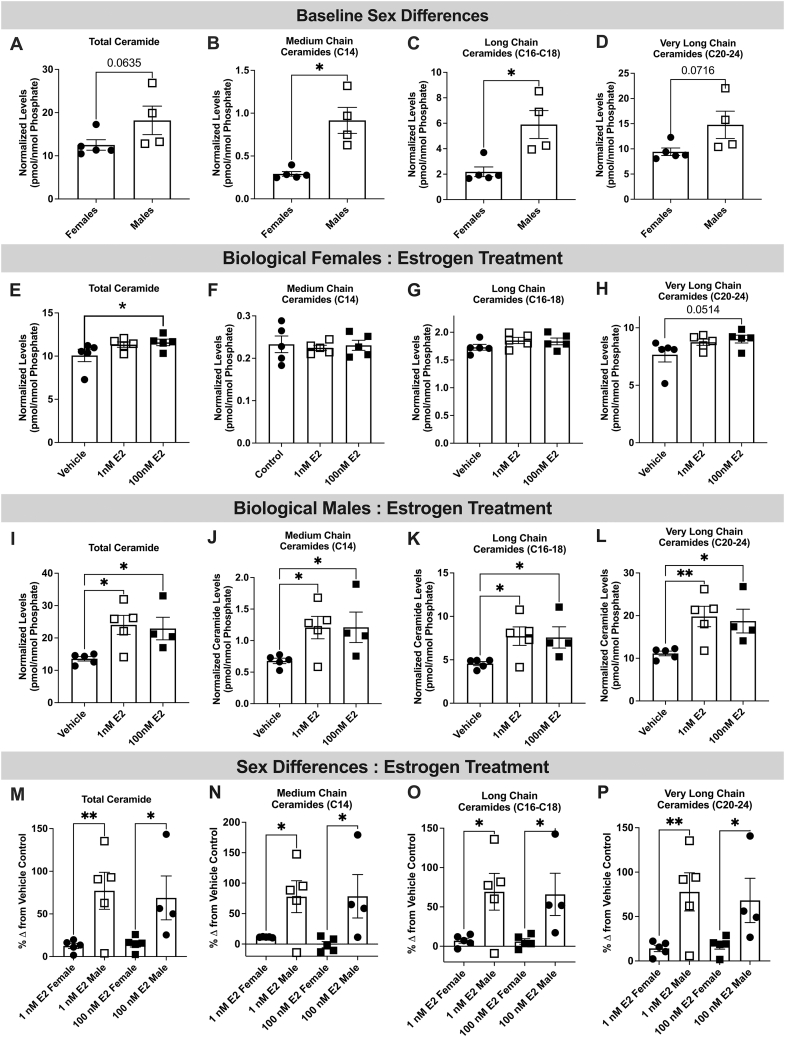
Endothelial Ceramide Content Is Higher Among Men Compared With Women at Baseline and Following Chronic Estrogen Exposure Normalized endothelial (A) total, (B) medium-chain, (C) long-chain, and (D) very-long chain ceramide levels in endothelial cells from men and women. (E) Total, (F) medium-chain, (G) long-chain, and (H) very-long chain ceramide levels in endothelial cells from women following vehicle (EtoH %vol/vol), 1 or 100 nmol/L 17β-estradiol (E2; 48 hours) treatment. (I) Total, (J) medium-chain, (K) long-chain, and (L) very-long chain ceramide levels in endothelial cells from men following vehicle, 1 and 100 nmol/L E2 treatment. Percent change from vehicle in (M) total, (N) medium-chain, (O) long-chain, and (P) very-long chain ceramide levels in endothelial cells from women compared with men following 1 and 100 nmol/L E2 treatment. Data are presented as mean ± SEM. (A-D) 2-tailed *t*-test, (E-P) 1-way analysis of variance ∗*P <* 0.05, ∗∗*P <* 0.01, ∗∗∗*P <* 0.001.

To evaluate whether chronic estrogen promotes ceramide formation in endothelial cells, sex-specific HUVECs were treated with 1 and 100 nmol/L of E2 for 48 hours and ceramide content measured using mass spectrometry. In untreated cells, ceramides, most notably medium and long-chain ceramides, were higher in cells from men compared with women (Figures 5A to 5D). Following 100 nmol/L treatment with E2, total ceramide content and very long-chain ceramide levels were increased in endothelial cells from women (Figures 5E to 5H), an effect not observed with the lower amount of E2 (1 nmol/L), whereas all chain-length ceramides were elevated following exposure to both doses of E2 in cells from men (Figures 5I to 5L). The percent increase in intracellular ceramides following 1 nmol/L and 100 nmol/L E2 treatment was higher among men compared with women (Figures 5M to 5P).

### Sex differences in endothelial ceramide to S1P ratio at baseline and following estrogen treatment

**Figure 6:**
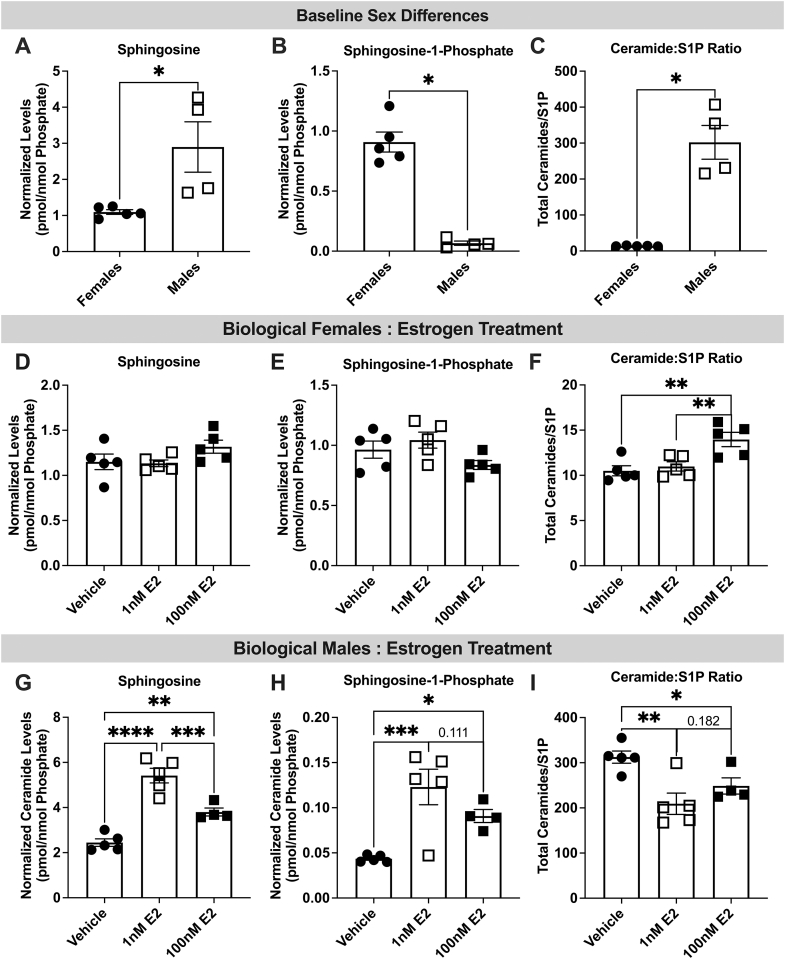
There Are Sex Differences in Ceramide to S1P Ratios at Baseline and Following Estrogen Treatment Normalized endothelial (A) sphingosine and (B) sphingosline-1-phosphate (S1P) levels as well (C) total ceramide to S1P ratio in control cells from men and women. (D) Sphingosine and (E) S1P levels as well (F) total ceramide to S1P ratio in endothelial cells from women following vehicle (EtoH %vol/vol), 1 or 100 nmol/L 17β-estradiol (E2) (48 hours) treatment. (G) Sphingosine and (H) S1P levels as well (I) total ceramide to S1P ratio in endothelial cells from women following vehicle, 1 or 100 nmol/L E2 treatment. Data are presented as mean ± SEM. (A-C) 2-tailed *t*-test, (D-I) 1-way analysis of variance ∗*P <* 0.05, ∗∗*P <* 0.01, ∗∗∗*P <* 0.001.

Because ceramide to S1P conversion is linked with NO production, we sought to determine whether chronic E2 exposure promotes ceramide accumulation with low conversion to S1P (ie, elevated ceramide to S1P ratio). Compared with untreated endothelial cells from women, men had higher levels of sphingosine—an intermediate lipid formed during ceramide to S1P conversion (Figure 6A). However, S1P levels were lower within cells from men compared with women (Figure 6B), and accordingly the ceramide to S1P ratio was higher among untreated cells from men (Figure 6C).

Following 1 and 100 nmol/L E2 treatment (48 hours), no significant changes in sphingosine or S1P levels were seen within cells from women (Figures 6D and 6E), although the total ceramide to S1P ratio was increased within cells treated with 100 nmol/L E2 (Figure 6F). Surprisingly, in cells from men, both sphingosine and S1P levels were increased following 1 and 100 nmol/L E2 treatment (Figures 6G and 6H). The level of sphingosine was significantly higher following 1 nmol/L compared with 100 nmol/L E2 treatment (Figure 6G), with a similar nonsignificant trend in S1P levels (Figure 6H). The ratio of ceramide to S1P was reduced with both 1 and 100 nmol/L E2, suggesting that cells from men had a high conversion of ceramides to S1P (Figure 6I).

### Suppression of mitochondrial oxidative stress prevents chronic estrogen-induced endothelial dysfunction

**Figure 7:**
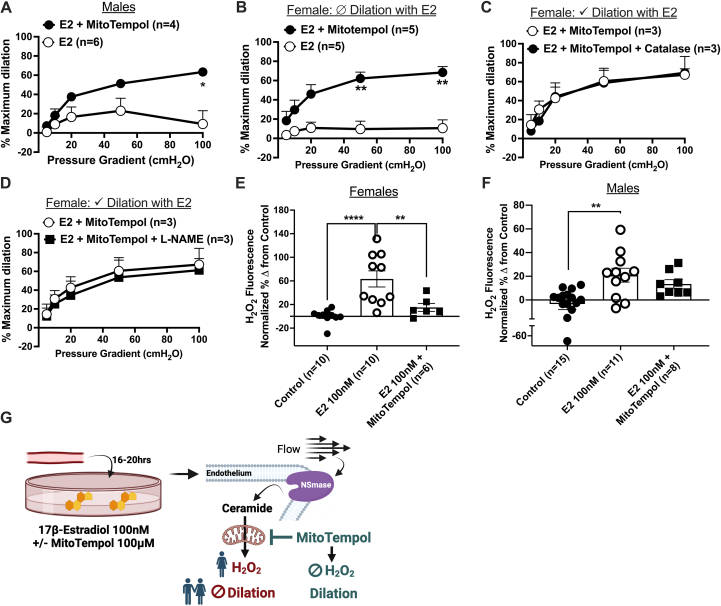
MitoTempol Protects Against Estrogen-Induced Endothelial Dysfunction (A) FID in vessels from men treated with 100 nmol/L 17β-estradiol (E2) (16-20 hours; n = 6) vs E2+MitoTempol (100 μmol/L; n = 4). (B) FID following E2+MitoTempol (100 μmol/L; n = 5) treatment within microvessels from women that had impaired dilation to flow following 100 nmol/L 17β-estradiol (E2) (16-20 hours; n = 5). Among vessels that maintained dilation to flow with 100 nmol/L E2 alone, dilation to flow following E2+MitoTempol (100 μmol/L; n = 3) treatment (C) +/− 30 minutes catalase (500 U/mL; n = 3), (D) +/− 30 minutes L-NAME (100 μmol/L; n = 3). H_2_O_2_ in HUVECs from (E) women (control n = 10, E2 n = 10, E2+MitoTempol n = 6) and (F) men (control n = 15, E2 n = 11, E2+MitoTempol n = 8) treated with 100 nmol/L 17β-estradiol (E2; 48 hours) +/− MitoTempol (100 μmol/L). (G) Schematic of results. Data are presented as mean ± SEM. (A-E) 2-way analysis of variance and (F-G) 1-way analysis of variance Holm-Sidak multiple comparisons test. ∗*P <* 0.05, ∗∗*P <* 0.01, ∗∗∗*P <* 0.001.

Chronic ceramide exposure is known to cause mitochondrial dysfunction in endothelial cells;^21^ therefore, we evaluated whether treatment with the mitochondrial antioxidant MitoTempol prevents estrogen-induced microvascular endothelial dysfunction in isolated arterioles. The addition of MitoTempol (100 μmol/L, 16-20 hours) during 100 nmol/L E2 treatment restored dilation (Figures 7A and 7B) to flow in microvessels from men as well as in vessels from women that did not dilate to flow following chronic E2. Among arterioles from women that maintained dilation but exhibited a switch to H_2_O_2_-mediated dilation following chronic E2 treatment, MitoTempol treatment preserved dilation in the presence of catalase (Figure 7C); yet, dilation was also maintained in the presence of L-NAME, suggesting that these vessels may rely on either H_2_O_2_ or NO (Figure 7D). The phenomenon is thought to represent a partial restoration of healthy vascular response.^20^ In isolated endothelial cells from women (Figure 7E), but not men (Figure 7F), MitoTempol+E2 treatment significantly diminished H_2_O_2_ production when compared with E2 alone. These results are schematized in Figure 7G. No significant differences in papaverine-induced dilation were seen across chronic estrogen treatment groups in women or men (Supplemental Figure 2).

## Discussion

The major findings of this study are 6-fold: 1) estrogen acutely promotes NO-mediated dilation in isolated arterioles from otherwise healthy women via the NSmase/ceramide/S1P pathway; 2) the extent of acute dilation to E2 is significantly lower among men compared with women; 3) in cultured endothelial cells, chronic estrogen leads to increased cellular H_2_O_2_ and ceramide production; 4) long-term exposure to estrogen impairs acute E2-mediated dilation and promotes H_2_O_2_ as opposed to NO production in response to acute ceramide; 5) there are notable sex differences in intracellular ceramide to S1P ratios both at baseline and following treatment with estrogen; and 6) estrogen-induced H_2_O_2_ formation in cells from men is lower despite exhibiting a lower ceramide to S1P ratio (ie, greater conversion of ceramide to S1P compared with baseline). Together, these data provides needed insight into the controversy regarding the NO-producing effects of estrogen and the increased cardiovascular disease risk with long-term exposure. Further, although ceramides are canonically associated with detrimental signaling and S1P is known to be more vasculoprotective, data here suggest that a basal level of ceramide formation is necessary for NO production in response to E2.

Clinically, CVD risk associated with estrogen therapy is often confounded by environmental risk factors such as smoking, lifestyle, comorbidities, and menopausal status. In this study, longer exposure to estrogen caused damage in both isolated human arterioles as well as in cultured human umbilical vein endothelial cells, models less influenced by environmental factors. Our previous work has demonstrated that microvessels from both younger (<40 years) and older (≥40 years) women exhibit a similar dysfunction following 16-20hrs of E29 therefore we did not differentiate our current results based on age or menopausal status. Here we have shown that modulation of the NSmase/ceramide pathway produces similar results across a heterogenous cohort of microvessels (women and men across the lifespan) implying that this signaling mechanism is conserved regardless of age or sex. More importantly, inhibition of NSmase as well as administration of a mitochondrial antioxidant during estrogen exposure prevented endothelial dysfunction, providing potential therapeutic targets for protecting cardiovascular health during estrogen therapy.

Interestingly, estrogen exposure diminished the ability of the hormone to elicit acute vasodilation in arterioles from women. These data alone may at least partly explain the controversial nature of estrogen within the vasculature. Although microvessels were exposed to 100 nmol/L of estrogen and the typical peak physiological dose in menstruating humans is 1 nmol/L, the precise amount of estrogen that endothelial cells are exposed to in vivo from estrogen therapy is unknown and likely much higher. Even so, acute administration of E2 doses of up to 10 μmol/L (as shown in Figure 1 and prior work^33^) have been shown to stimulate NO production in both cells and isolated blood vessels. The fact that acute E2 signaling is altered and NO signaling is impaired following chronic exposure begs the question of whether the vasculoprotective properties of estrogen are diminished in the setting of continuous administration. This may suggest that the natural fluctuating nature of the hormone provides benefit as opposed to continuous, chronic exposure during estrogen therapy that may lead to increased CVD risk.^14^ Numerous studies have linked elevated ceramide levels to increased risk for major adverse cardiovascular events,^34^^,^^35^ oxidative vascular damage,^36^, ^37^, ^38^ and microvascular dysfunction.^39^ Our results suggest that chronic estrogen therapy can increase aberrant ceramide accumulation in human microvascular endothelial cells, triggering microvascular dysfunction. Further, given our results showing that inhibition of chronic NSmase-mediated ceramide formation can protect against chronic estrogen-induced microvascular dysfunction, the use of ceramide inhibitors may offer a potential therapeutic strategy to protect vascular health among those utilizing supplemental estrogen (eg, hormone replacement therapy, oral contraceptives).

The response to acute ceramide was also altered in vessels exposed to chronic estrogen as arterioles produced H_2_O_2_ as opposed to NO, an effect also observed in arterioles from patients with CAD. Treatment with either an inhibitor of NSmase or a mitochondrial antioxidant prevented this, a similar result observed in vessels from individuals with CAD.^21^ Inhibition of NSmase, however, was not successful in preventing the loss of acute E2-induced signaling and suggests that estrogen’s ability to activate NSmase-dependent ceramide formation is impaired following chronic E2 treatment. There are many reasons that may account for this, including but not limited to internalization of membrane localized estrogen-receptors^40^ and/or changes in the expression of specific receptors themselves. Understanding the mechanisms contributing to the desensitization of acute estrogen signaling in human arterioles following chronic estrogen treatment, including the role of the estrogen receptors, is an important avenue of future exploration.

Key biological sex differences were noted in this study. First, the acute response to estrogen (dilation) was significantly reduced in arterioles from men compared to women. This is not surprising given prior work showing that endothelial expression of estrogen receptors is significantly lower among men compared with premenopausal women,^9^ and studies have linked acute estrogen NO-producing effects to the availability of membrane-bound estrogen receptors.^41^ Endothelial expression of endothelial NO synthase is also lower in men compared with women, which may limit estrogen’s ability to acutely promote NO production.^42^ Furthermore, our results show that compared with women, endothelial cells from men have significantly higher ceramide levels, including the more damaging long-chain ceramides, and lower levels of S1P. These data align with other studies that have demonstrated lower plasma levels of S1P in men vs premenopausal women.^16^ Knowledge gaps exist regarding sex differences with respect to ceramide pathway enzymes. For instance, it remains unknown whether sex alone determines expression or activity of sphingomyelinases, ceramide synthases, or ceramidases, which are key in determining the cellular composition of sphingolipids. It is possible that the unique, sex-specific sphingolipid balance within endothelial cells partially explains sex differences regarding CVD risk.

In this study and prior work,^9^ 2 unique phenotypes were identified within arterioles from otherwise healthy women (<1 risk factor for coronary artery disease) following chronic estrogen exposure. One cohort maintained dilation to flow but exhibited a switch from NO- to H_2_O_2_-mediated dilation, while the other had severe dysfunction as evidenced by a reduction in vasodilatory capacity to shear. These differences are not explained by age or BMI, which was in line with prior work showing that human microvascular response to flow among otherwise healthy women depends on NO up to 80 years of age/postmenopause.^9^^,^^43^ Thus, other factors beyond menopausal status or age are likely responsible for this difference. However, regardless of the extent of dysfunction, inhibition of NSmase-mediated ceramide formation restored vascular function. An intriguing question is whether the extent of ceramide accumulation and/or ceramide-induced H_2_O_2_ is higher in the cohort that experiences a reduction in max dilation. The ability to discern populations that are more susceptible to microvascular dysfunction with estrogen treatment may prove critical in preventing future CVD and future work will focus on elucidating potential drivers of this phenotypic difference within women.

### Study limitations

Important limitations of this study should be noted. The use of HUVECs for our cell culture model is not a true representation of microvascular endothelial cells, although they are a reliable, reproducible, and cost-effective model; the availability and reproducibility of robust sex-specific microvascular endothelial cells are currently limited. Arterioles were collected from a patient population that is reflective of the local surgical population at our institution. As such, there is limited racial diversity. It is also fairly uncommon to receive discarded surgical specimens from women of trans experience who have taken gender-affirming estrogen therapy. For this study we utilized arterioles from men as a surrogate for understanding the potential mechanisms of microvascular dysfunction in this patient population, although this strategy does allow for identifying the direct effects of estrogen on those assigned male at birth without any confounding influences from social/psychological factors that are known to influence cardiovascular health among women of trans experience. Furthermore, given the limited number of vessels one can acquire from each patient sample, we were limited in terms of the number of experiments per sample. As such, we were unable to determine whether a second phenotype exists for the acute response to estrogen (dilation). Another important limitation is the lack of information regarding the use of hormones, menopausal status, or stage of menstrual cycle among our patients. Information on other noncardiac comorbidities, clinical characteristics, and medical usage is also limited for discarded samples. All vessels undergo an extensive wash protocol overnight, which likely limits the influence of pharmacological agents in the system, although, any chronic changes within the microvessels themselves cannot be controlled for. Nonetheless, the fact that we were able to identify a conserved mechanism of acute and chronic estrogen signaling in our diverse human arterioles highlights the critical role of this pathway in influencing microvascular endothelial health among those with heterogenous genetic and environmental backgrounds.

## Conclusions

This study provides critical insights into the complex and sex-specific microvascular effects of estrogen, particularly its role in modulating NO-mediated dilation through the NSmase/Ceramide/S1P signaling pathway. The findings highlight key differences between males and females in both acute and chronic responses to estrogen and challenges the traditional dichotomy of ceramide as purely harmful and S1P as solely protective, suggesting instead that a balanced ceramide:S1P ratio is essential for optimal endothelial health. These findings deepen our understanding of estrogen's dual vascular effects and may help explain the paradoxical increase in cardiovascular disease risk observed with long-term hormone exposure.

## Funding Support and Author Disclosures

This work was funded by American Heart Association (AHA) NHLBI R01HL160752 ( Dr. Freed) and predoctoral fellowship grant 909315 to (Dr. SenthilKumar).

Perspectives **COMPETENCY IN MEDICAL KNOWLEDGE:** We report that estrogen stimulates microvascular endothelial ceramide production via the neutral sphingomyelinase pathway in a sex-dependent manner. This observation is critical in understanding how natural sex hormones influence human microvascular function.

**TRANSLATIONAL OUTLOOK:** While it is well-established that coronary microvascular dysfunction is twice as likely to occur in women than men, mechanisms driving this clinical observation remain unknown. Understanding differences in human microvascular function based on sex will allow for the development of more precise treatment options and potentially create new avenues to prevent coronary microvascular dysfunction.
