# Supplementary material for: Estrogen Influences Human Microvascular Endothelial Function Via Sex-Specific Regulation of Sphingolipids
Source: JACC Basic Transl Sci. 2025 Oct 25;11(1):101389. doi: 10.1016/j.jacbts.2025.101389 (PMC12902239; doi:10.1016/j.jacbts.2025.101389)
Supplement: Supplementary Figures 1 and 2 [file mmc1.docx]

## **Supplemental Figures**

***Supplemental Figure 1.*** *Dilation to acetylcholine (10^-9^ to 10^-4^, 2 mins) in vessels from biological females treated with 100nM E2 for 16-20hrs. Data are presented as mean ± SEM. Two-way repeated measures ANOVA.*

*
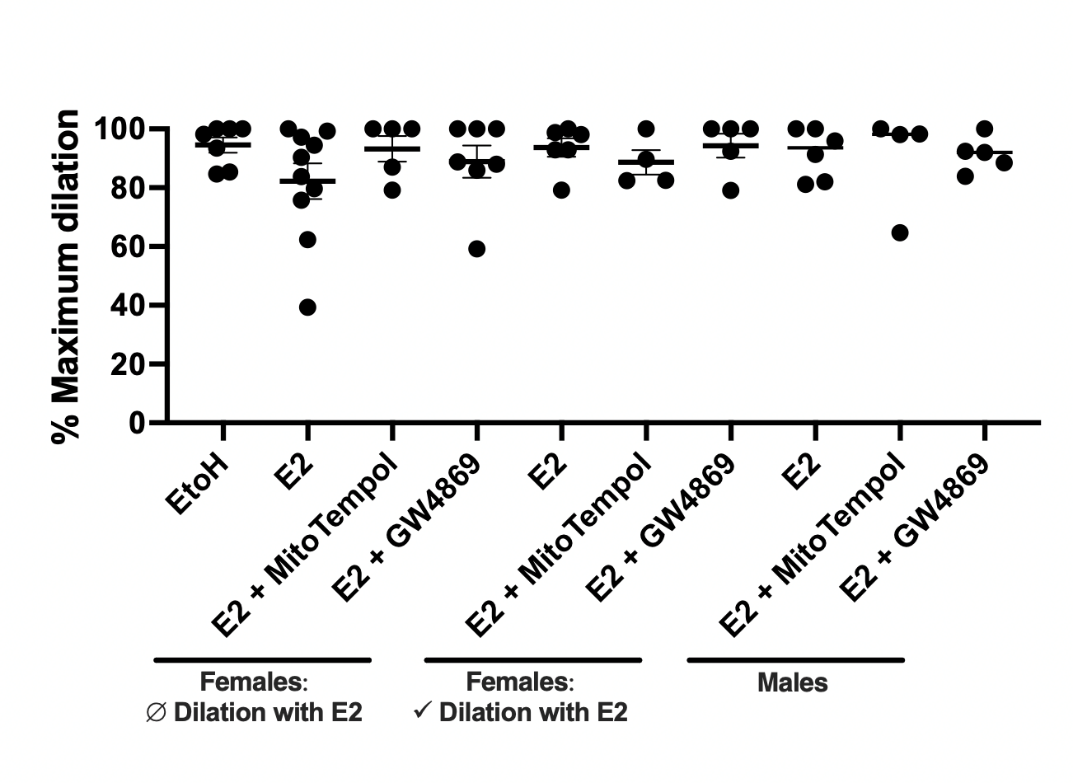
*

***Supplemental Figure 2****. Papaverine induced dilation in vessels from biological females and males treated with chronic 100nM E2 +/- 100 uM Mitotempol or +/- 4uM GW4869. Data are presented as mean ± SEM. One way ANOVA.*
